# The Epidemiology of Healthcare‐Acquired Respiratory Syncytial Virus Infection Among Hospitalised Paediatric Patients: a Systematic Review and Meta‐Analysis

**DOI:** 10.1111/irv.70169

**Published:** 2025-10-14

**Authors:** B. Wahi‐Singh, P. Wahi‐Singh, C. Lau, A. Buckle, P. Manzoni, H. Nair

**Affiliations:** ^1^ Centre for Global Health, Usher Institute University of Edinburgh Edinburgh United Kingdom; ^2^ Edinburgh Medical School Edinburgh United Kingdom; ^3^ Royal Hospital for Children and Young People NHS Lothian Edinburgh United Kingdom; ^4^ Department of Public Health and Pediatric Sciences University of Torino School of Medicine Turin Italy; ^5^ Division of Paediatrics and Neonatology Degli Infermi Hospital Ponderano Italy; ^6^ National Vaccine Innovation Platform, School of Public Health Nanjing Medical University Nanjing China; ^7^ MRC/Wits Rural Public Health and Health Transitions Unit (Agincourt), School of Public Health University of the Witwatersrand Johannesburg South Africa

**Keywords:** epidemiology, healthcare‐acquired infection, incidence, meta‐analysis, nosocomial, paediatric, respiratory syncytial virus, RSV

## Abstract

**Background:**

Hospital‐acquired respiratory syncytial virus (HA‐RSV) infections pose a substantial risk to hospitalised children, previously described as composing a fifth of RSV‐related deaths worldwide. Despite this, the epidemiology of HA‐RSV remains under‐characterised, with limited meta‐analytical evidence quantifying its incidence, morbidity and mortality.

**Methods:**

We conducted a systematic review and meta‐analysis of English language papers published between January 1975 and March 2024 searching EMBASE, MEDLINE and CABI Global Health. We included studies with primary data on paediatric HA‐RSV cases among either all patients, patients with RSV or patients with healthcare‐acquired infections (HAIs). Outbreak reports were excluded for the purposes of this analysis. Using random‐effects meta‐analyses, we synthesised the HA‐RSV incidence rate (IR) and mortality rate (MR) among patient groups, reported as cases and deaths per 1000 person‐years respectively. HA‐RSV cumulative incidence and case‐fatality rate (CFR) are also reported as percentages. The Joanna‐Briggs Institute critical appraisal tool was used for quality assessment.

**Results:**

Twenty‐seven studies from 11 countries were included. The pooled HA‐RSV IR among all paediatric patients was 10.86 cases (95% confidence interval, 3.83–17.89) per 1000 person‐years. The MR was 11.34 (5.57–17.11) deaths per 1000 person‐years, and the pooled CFR was 13.30% (3.21%–23.40%). HA‐RSV comprised 15.57% of RSV hospitalisations and 22.48% of all HAIs.

**Conclusions:**

HA‐RSV is a serious and under‐recognised cause of morbidity and mortality in hospitalised paediatric patients, with significantly higher mortality than community‐acquired RSV. These findings underscore the need for strengthened infection control, standardised diagnostic criteria and targeted preventative strategies to mitigate its impact globally.

## 1 Introduction

Respiratory syncytial virus (RSV) is one of the leading causes of lower respiratory tract infections and deaths in children [1]. While substantial progress has been made in the systematic analysis of paediatric community‐acquired RSV (CA‐RSV) [2], the burden and impact of healthcare‐associated RSV (HA‐RSV) are not fully appreciated. RSV infection is a significant risk for hospitalised children, particularly neonates and immunocompromised patients [3]. Its infectious profile is such that it can be easily transmitted in healthcare environments, with large droplet viral viability for up to 5 hours on gloves and up to 7 hours on surfaces such as countertops [4, 5]. Additionally, these infections may present atypically, with nonspecific signs, signs of cardiopulmonary decompensation or as an influenza mimic, increasing diagnostic challenges [3].

Many studies have demonstrated significant differences in outcomes of CA‐RSV and HA‐RSV [6, 7], and cost benefits of infection‐control measures to reduce HA‐RSV have been shown as well [8]. While fewer have analysed differences in morbidity, some studies have also suggested differences in paediatric intensive care unit (PICU) length of stay and ventilator usage [9].

HA‐RSV has been previously reported to comprise a fifth of RSV‐related deaths worldwide [10]; however, understanding the implications of this is difficult without estimating the incidence of HA‐RSV cases and the global HA‐RSV disease burden.

So far, there have been only two previous literature reviews conducted on HA‐RSV: one, a systematic review on HA‐RSV, not limited to paediatric populations; the other, a literature search limited to neonatal intensive care unit (NICU) HA‐RSV outbreaks [11, 12]. Neither was a meta‐analysis. With this study, we thus expand on the specific risks in paediatric HA‐RSV. Along with previous estimates of CA‐RSV global disease burden [2], our analyses will provide a greater understanding of HA‐RSV epidemiology to help inform policy decisions on an international scale. Furthermore, defining the incidence of HA‐RSV among healthcare‐acquired infections (HAIs) can also aid institutions when considering nosocomial disease prevention priorities on a local scale.

## 2 Materials and Methods

### 2.1 Search Strategy and Selection Criteria

We conducted a systematic literature review (registered with PROSPERO: CRD42022314323) to identify studies reporting data on neonatal and paediatric HA‐RSV cases among either: all paediatric patients, paediatric patients with RSV or paediatric patients with HAIs. We searched EMBASE, MEDLINE and the CABI Global Health databases for relevant articles published between January 1975 and March 2024, using a tailored search strategy developed and applied to each database (Tables S1–S3). Selection criteria for studies are as follows:

Inclusion Criteria

- Reporting data for HA‐RSV infections in children under 18 years old as a primary infection AND
- Reporting data on incidence rate (IR), hospitalisation rate (HR), in‐hospital case‐fatality rate (CFR) or risk factors for HA‐RSV infections in children under 18 years old AND
- Reporting data for at least 12 consecutive months, except for in‐hospital CFR or studies reporting data for the full RSV season where seasonality is well established and documented.

Exclusion Criteria

- RSV infection was not clearly defined, or the case definition is not consistently applied OR
- IR, hospitalisation rate, in‐hospital CFR or risk factors for HA‐RSV infections in children under 18 years old was estimated by modelling techniques with no primary data on RSV infection (e.g., regression models of viral activity with nosocomial infection time series and mathematical models) OR
- RSV diagnosis based on serology alone OR
- Only including infants who received RSV prophylaxis OR
- Case reports, case series, outbreak reports, editorials or systematic reviews.

All studies identified by the literature search were screened firstly by title and abstract, followed by full‐text screening. Where papers may have been relevant, but further clarification was needed to determine inclusion, the review team reached out to study authors for further information. For all included studies, general information such as study period, location and country, subject age and case definition for HA‐RSV infection was collected using a predesigned assessment form. Data screening and extraction were conducted independently by BWS and PWS, with any inconsistencies resolved through discussions among members of the review team.

Due to the lack of consistency between definitions of RSV and what cases should be considered ‘hospital‐acquired,’ studies were included so long as they did not solely rely on serology for diagnosis and consistently applied their definitions of HA‐RSV and CA‐RSV. Definitions of HA‐RSV were extracted and have been displayed in Table 1 in the Results section.

**TABLE 1:** Study characteristics of included studies.

Author	Year	Study design	Location	Age group (for paediatric patients)	HA‐RSV definition	Study quality (%)
Aikphaibul	2021	Retrospective cohort	Bangkok, Thailand	≦ 5y	RSV‐LTRI diagnosis > 72 h post‐admission	95
Alan	2016	Prospective cohort	Turkey	Unspecified (presumed ≦ 6 m)	Signs/symptoms ≧ 48 h post‐admission	55
Asanathong	2017	Retrospective cohort	Bangkok, Thailand	≦ 18y	Signs/symptoms ≧ 48 h post‐admission	55
Asner	2013	Retrospective cohort	Toronto, Canada	≦ 18y	Signs/symptoms > 72 h post‐admission	91
Chemaly	2014	Retrospective cohort	Houston, Texas, USA	≦ 18y	Signs/symptoms > 96 h post‐admission	85
Corten	2020	Retrospective cohort	Cape Town, South Africa	≦ 18y	LRTI in discharge diagnoses but not primary diagnosis	65
Ehlken	2005	Prospective cohort	Germany	≦ 3y	Signs/symptoms > 48 h post‐admission	70
Feldman	2022	Case–control	Orange County, California, USA	≦ 21y	RSV laboratory diagnosis ≧ 48 h post‐admission	60
Fodha	2004	Prospective cohort	Sousse, Tunisia	≦ 35d	RSV diagnosis > 8 days post‐admission or ≦ 5 days post‐discharge	89
Gagneur	2002	Prospective cohort	Quebec, Canada	Unspecified (presumed ≦ 18 y)	Negative specimen on admission with positive viral detection later	61
Gagneur	2008	Prospective cohort	Brest, France	Unspecified (presumed ≦ 18 y)	Negative specimen on admission with positive viral detection later	56
Krasinski	1990	Prospective cohort	New York City, New York, USA	Unspecified (presumed ≦ 18 y)	RSV‐LTRI diagnosis > 4 days post‐admission	70
Lo	2013	Retrospective cohort	Boston, Massachusetts, USA	Unspecified (presumed ≦ 18 y)	RSV laboratory diagnosis > 3 days post‐admission	91
Macartney	2000	Prospective cohort	Philadelphia, Pennsylvania, USA	Unspecified (presumed ≦ 18 y)	RSV diagnosis ≧ 6 days post‐admission or ≦ 5 days post‐discharge	75
Mackie	2001	Prospective cohort	Glasgow, UK	≦ 2y	RSV laboratory diagnosis > 7 days post‐admission	67
Madge	1992	Prospective cohort	Glasgow, UK	≦ 2y	RSV laboratory diagnosis > 7 days post‐admission	91
Moler	1992	Retrospective cohort	Ann Arbor, Michigan, USA	Unspecified (presumed ≦ 18 y)	RSV laboratory diagnosis > 4 days post‐admission for non‐febrile illness	77
Ozen	2023	Prospective cohort	Adana, Turkey	≦ 16y	Signs/symptoms ≧ 3 days post‐admission	65
Petrie	2024	Retrospective cohort	Michigan, USA	<18y	RSV laboratory diagnosis > 6.3 days post‐admission	86
Rodriguez‐Auad	2012	Retrospective cohort	Mexico City, Mexico	≦ 18y	RSV laboratory diagnosis ≧ 6 days post‐admission or < 5 days post‐discharge	75
Ruenglerdpong	2023	Case–control	Bangkok, Thailand	≦ 5y	RSV laboratory diagnosis ≧ 6 days post‐admission	85
Simon	2008	Prospective cohort	Germany	Unspecified (presumed ≦ 18 y)	Signs/symptoms ≧ 5 days post‐admission	82
Thorburn	2008	Prospective cohort	Liverpool, UK	Unspecified (presumed ≦ 18 y)	RSV laboratory diagnosis ≧ 5 days post‐admission	75
Thorburn	2012	Prospective cohort	Liverpool, UK	Unspecified (presumed ≦ 18 y)	RSV laboratory diagnosis ≧ 5 days post‐admission	100
Vayalumkal	2009	Prospective cohort	Canada	<18 y	Signs/symptoms ≧ 72 h post‐admission	75
Weigl	2002	Retrospective cohort	Kiel, Germany	≦ 16 y	Signs/symptoms ≧ 48 post‐admission for non‐ARI illness	68
Wrotek	2020	Retrospective cohort	Warsaw, Poland	Unspecified (presumed ≦ 1 y)	Signs/symptoms ≧ 48 h post‐admission or ≦ 3 post‐discharge	72

Quality assessment of all studies was conducted independently by two reviewers using the Joanna‐Briggs Institute (JBI) critical appraisal scoring tool to assess the quality of included studies [13]. For each study, we defined quality using the following formula based on responses to questions assessing for high quality: (‘Yes’ + 0.5*(‘Unclear’))/Applicable Questions. A score of 0.7 or greater was considered high quality, 0.5–0.7 medium quality and < 0.5 low quality.

### 2.2 Statistical Analysis

The primary outcomes were (1) HA‐RSV IR among all patients, measured as HA‐RSV cases per 1000 person‐years and (2) deaths among children with HA‐RSV, measured as mortality rate (MR). Secondary outcomes included the HA‐RSV CFR, measured as HA‐RSV deaths per 1000 person‐years, and cases of paediatric HA‐RSV, measured as the cumulative incidence (CI). HA‐RSV CI was also reported among patients with RSV, among patients with any HAI and among patients with any nosocomial acute respiratory infection (HA‐ARI). Where at least two studies with relevant data and with at least one event were available, we also conducted analyses among patients admitted to an intensive care unit (ICU).

Data were synthesised using random‐effects meta‐analyses using a frequentist framework. A continuity correction was applied for zero‐event studies, ensuring stable variance estimation and inclusion in the meta‐analysis [14]. Models with a normal likelihood for the log‐transformed IR data were used. The restricted maximum likelihood estimator was applied to estimate between‐study heterogeneity. Sensitivity analysis with leave‐one‐out cross‐validation was employed to assess the impact of individual studies for each outcome. Statistical analysis was performed using r software (version 4.2.2) and the metafor package (version 4.6.0).

## 3 Results

### 3.1 Systematic Review

**FIGURE 1:**
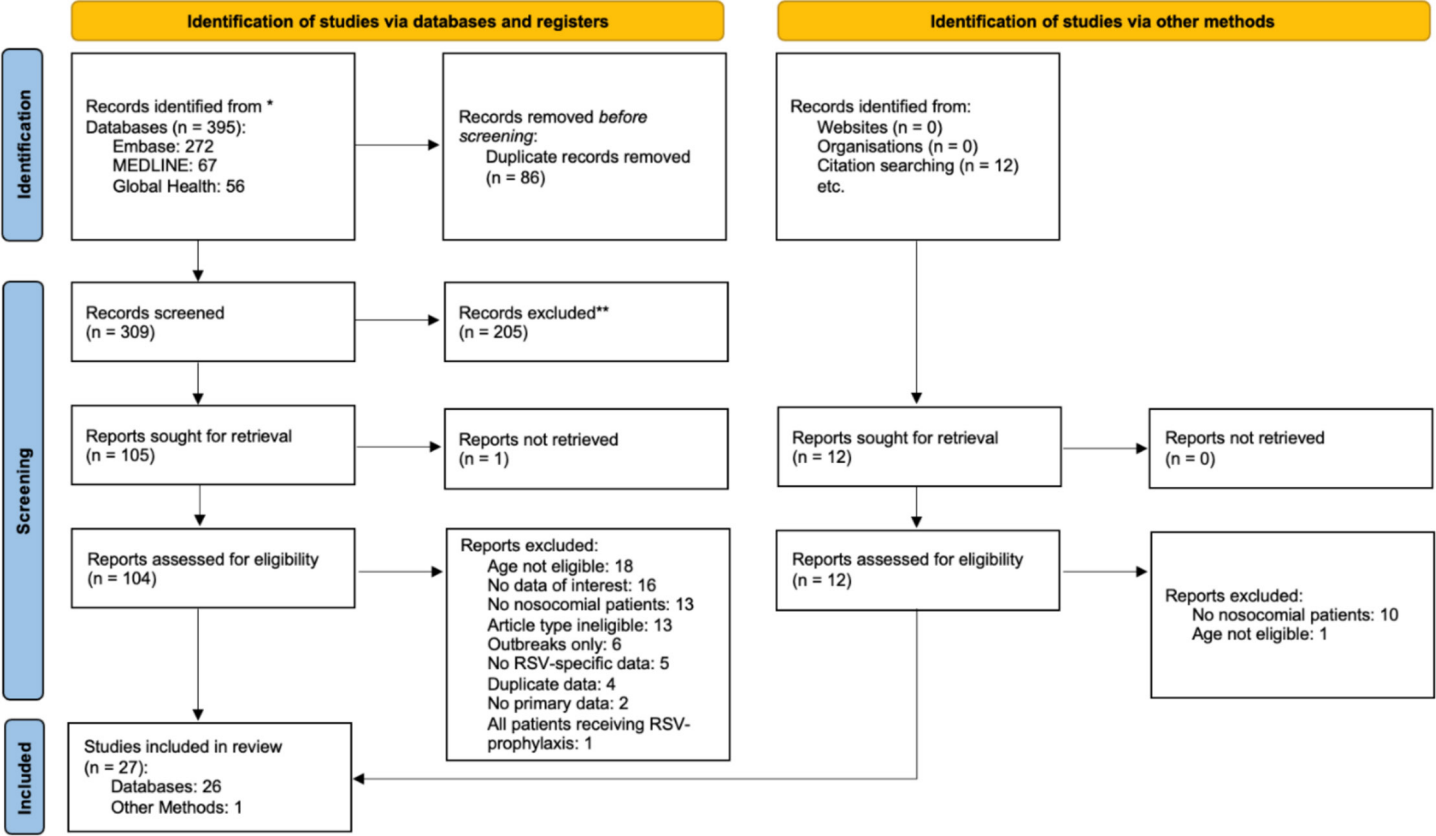
PRISMA flow diagram of included studies.

Four hundred seven records were identified: 395 through systematic review of the literature and 12 through citation searching. Of these, 116 were considered for full‐text review, and 27 studies from 11 countries met selection criteria and were included in the analysis of at least one primary outcome (Figure 1). The studies were distributed by WHO region in the following manner: 11 region of the Americas, 11 European region, 3 South‐East Asian region, 1 African region and 1 Eastern Mediterranean region [13]. Nineteen studies were from high‐income countries, seven from upper‐middle income countries and one from a lower‐middle income country [15]. Nineteen studies were from developed countries and eight from developing countries [16]. Eighteen studies were rated high‐quality (Tables S4 and S5). Characteristics of each included study are listed in Table 1. Of the 27 studies eligible for at least one outcome, 15 were eligible for analysis of HA‐RSV IR in a general population, 6 in patients with an ARI, 6 in patients at ward‐level care, 4 in patients admitted to the NICU or PICU and 3 in patients < 3 years old. Thirteen studies were eligible for analysis for HA‐RSV CFR and 13 for HA‐RSV MR. Studies included in analyses of primary outcomes are shown in Table 2.

**TABLE 2:** Included studies for primary outcomes.

Author	Year	CI (all patients)	IR (all patients)	CI (RSV patients)	CI (HAI patients)	CFR	MR
Aikphaibul	2021	No	No	Yes	No	Yes	Yes
Alan	2016	Yes	Yes	Yes	No	Yes	Yes
Asanathong	2017	Yes	Yes	No	Yes	No	No
Asner	2013	No	No	Yes	No	Yes	Yes
Chemaly	2014	No	No	Yes	No	Yes	Yes
Corten	2020	Yes	Yes	Yes	Yes	No	No
Ehlken	2005	No	Yes	No	No	No	No
Feldman	2022	No	No	No	Yes	No	No
Fodha	2004	Yes	Yes	Yes	No	No	No
Gagneur	2002	Yes	Yes	Yes	Yes	No	No
Gagneur	2008	Yes	Yes	Yes	Yes	No	No
Krasinski	1990	No	Yes	No	No	No	No
Lo	2013	Yes	Yes	Yes	Yes	Yes	Yes
Macartney	2000	No	Yes	No	No	No	No
Mackie	2001	Yes	No	Yes	No	No	No
Madge	1992	Yes	Yes	Yes	No	No	No
Moler	1992	No	No	Yes	No	Yes	Yes
Ozen	2023	Yes	Yes	Yes	Yes	No	No
Petrie	2024	Yes	Yes	No	Yes	Yes	Yes
Rodriguez‐Auad	2012	Yes	Yes	Yes	No	Yes	Yes
Ruenglerdpong	2023	No	No	No	No	Yes	Yes
Simon	2008	No	No	Yes	No	Yes	Yes
Thorburn	2008	No	No	Yes	No	Yes	Yes
Thorburn	2012	No	No	Yes	No	No	No
Vayalumkal	2009	No	No	No	Yes	Yes	Yes
Weigl	2002	Yes	Yes	Yes	Yes	No	No
Wrotek	2020	No	No	Yes	No	No	No

### 3.2 Hospital‐Acquired Respiratory Syncytial Virus Incidence Rate and Mortality Rate

**FIGURE 2:**
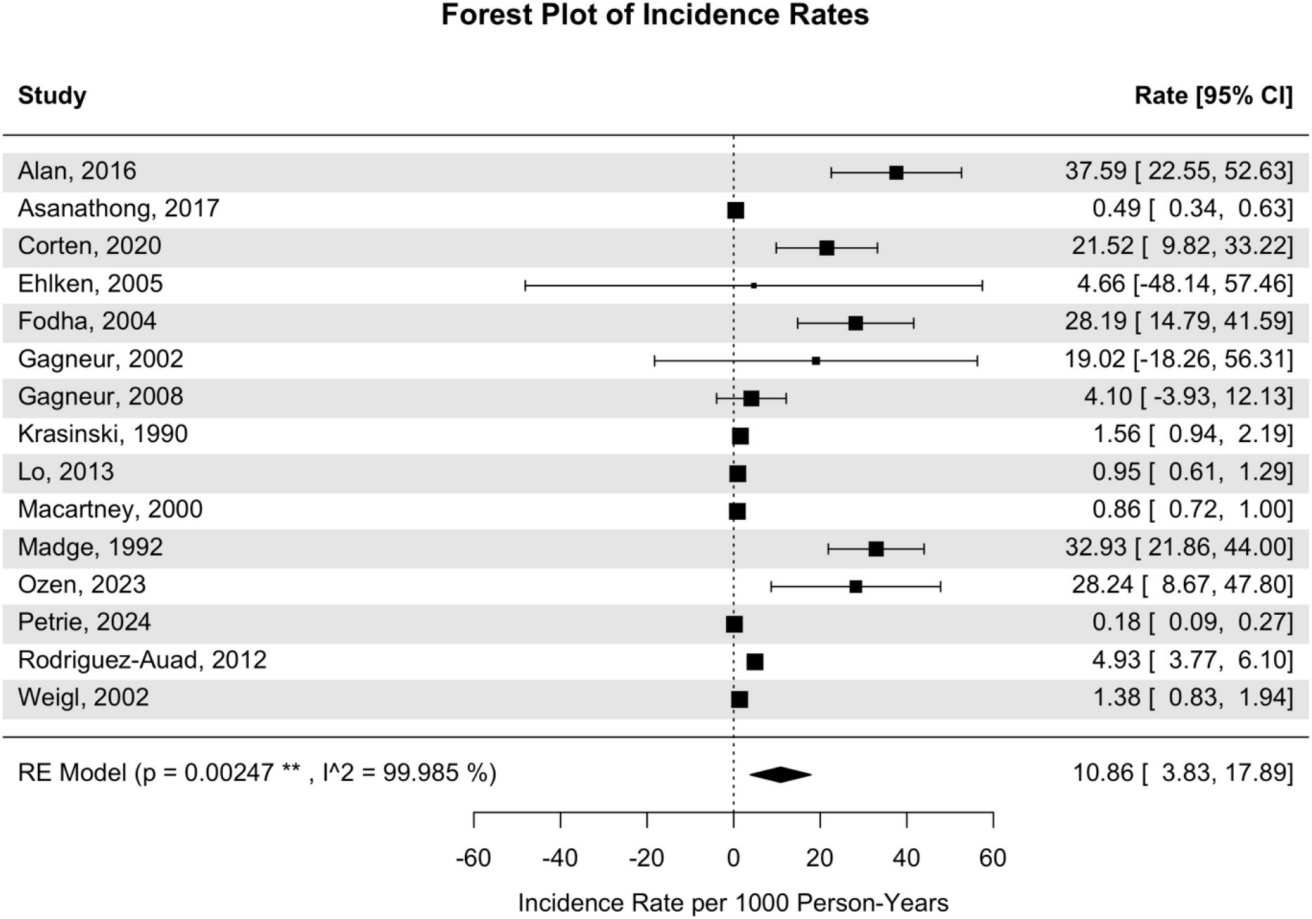
Forest plot of incidence of HA‐RSV cases among paediatric patients per 1000 person‐years.

**FIGURE 3:**
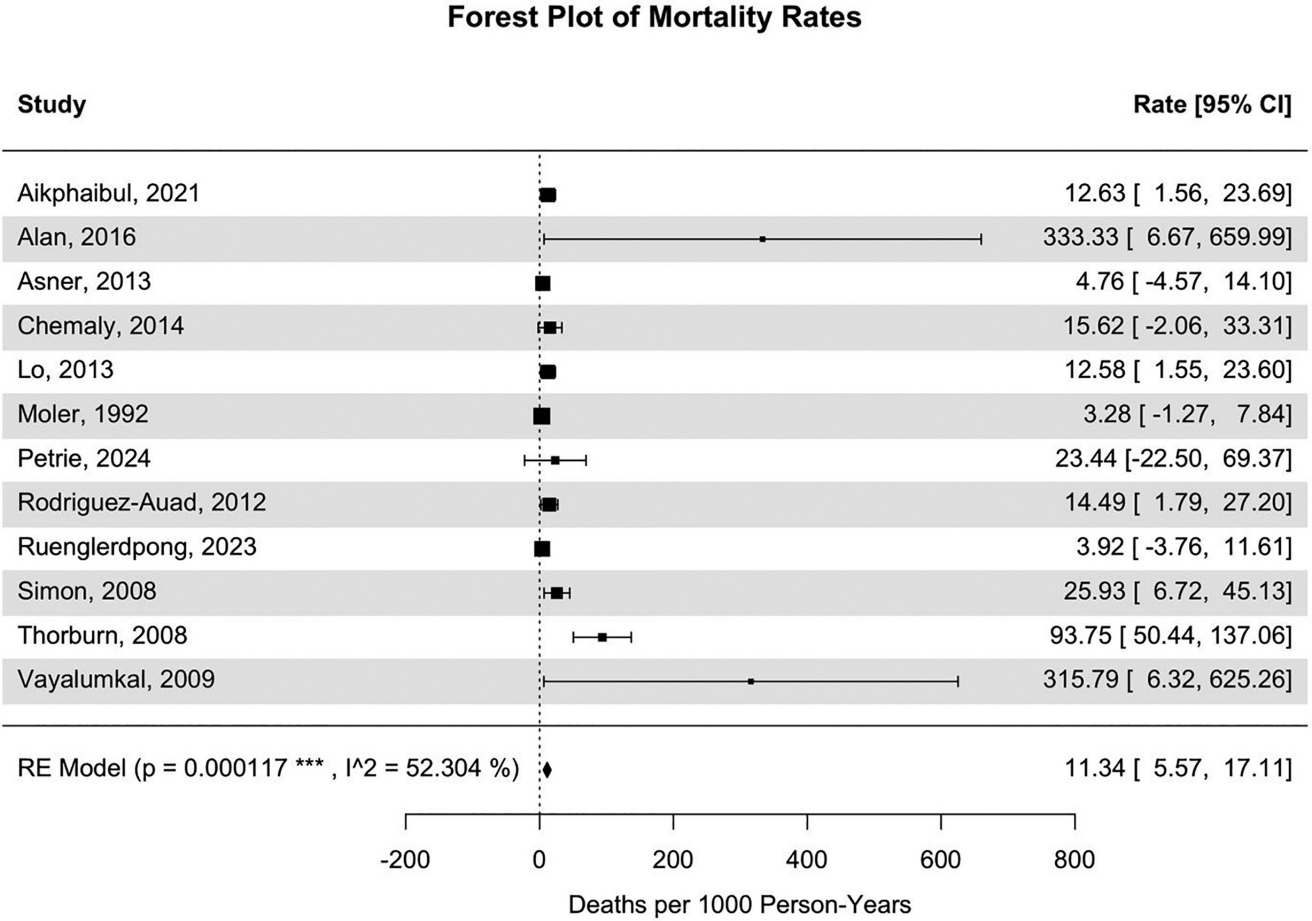
Forest plot of HA‐RSV deaths among paediatric patients per 1000 Person‐Years.

Fifteen studies from 10 countries (representing a total 535,014.3 person‐years) were included in the analysis of HA‐RSV incidence among all paediatric patients. HA‐RSV IR in all paediatric patients was 10.86 (3.83–17.89) cases per 1000 person‐years (Figure 2), with Egger's regression test yielding a significant result (*t* = 4.3796, *p* = 0.0007). HA‐RSV IR was 11.96 (2.30–21.63) cases per 1000 person‐years in patients with an ARI (total 134,972.3 person‐years) and was 30.34 (21.91–38.76) cases per 1000 person‐years in patients < 3 years of age (total 101,635.5). HA‐RSV IR was 21.58 (4.17–38.98) cases per 1000 person‐years in patients admitted to the NICU or PICU (total 1218.4 person‐years) but was only 13.12 (1.19–25.05) in patients at ward‐level care (total 118,195.2 person‐years, meta‐analyses in Supplementary Figures S1 and S2). The synthesised HA‐RSV MR based on 2933.833 person‐years from 13 studies in seven countries was 11.34 (5.57–17.11) deaths per 1000 person‐years (Figure 3). Sensitivity analyses of HA‐RSV IR and MR with studies rated high‐quality are available in Supplementary Figures S8 and S9.

### 3.3 Hospital‐Acquired Respiratory Syncytial Virus Cumulative Incidence and Case Fatality Rate

HA‐RSV CI outcomes are displayed in Table 3. The CFR, based on 553 patients from 13 studies in seven countries, was 13.30% (3.21%–23.40%). The full forest plots for HA‐RSV CI outcomes are available in Supplementary Figures S3–S5.

**TABLE 3:** Cumulative incidence of HA‐RSV among various patient populations.

Population denominator	Cumulative incidence of HA‐RSV (95% confidence interval)	Studies included	Total patients in analysis	*I* ^2^	*τ* ^2^
All patients	1.19 (0.59 to 1.80)	13	67,812	99.41%	0.0001
Patients < 3 y of age	2.43 (−1.00 to 5.85)	3	4526	99.07%	0.0009
Patients with an ARI	5.96 (1.35 to 10.57)	5	6842	99.21%	0.0025
Patients admitted to a PICU or NICU	1.42 (0.14 to 2.69)	4	1741	74.83%	0.0001
Patients with RSV	15.57 (9.34 to 21.80)	19	12,841	99.77%	0.0168
Patients < 3 y of age with RSV	9.95 (−5.36 to 25.26)	4	2215	99.78%	0.0233
Patients with RSV admitted to a PICU or NICU	16.67 (1.93 to 31.42)	5	685	95.74%	0.0204
Patients with RSV admitted to a PICU	20.21 (−0.36 to 40.78)	4	435	78.89%	0.0326
Patients with any HAI	22.48 (9.54 to 35.42)	10	1674	98.45%	0.0411
Patients with any HA‐ARI	13.78 (7.40 to 20.15)	5	866	83.48%	0.0037
Patients with any HAI admitted to a PICU or NICU	12.97 (−0.03 to 25.98)	4	156	88.99%	0.0149
Patients with any HAI admitted to a PICU	16.71 (5.95 to 27.48)	4	105	57.86%	0.0069

## 4 Discussion

Our meta‐analysis including data from 15 studies covering 826,713.6 person‐years demonstrates that HA‐RSV is an under‐appreciated yet serious and widespread problem among hospitalised neonatal and paediatric patients, with 10.86 new cases per 1000 person‐years. Analysis of CI found it comprises 15.57% of RSV hospitalisations (12,841 patients over 19 studies) and 1.09% of all hospitalisations (68,176 patients over 15 studies). HA‐RSV further accounts for nearly 1 in 4 paediatric HAIs (1658 patients over 10 studies), making it particularly significant when considering these. Markedly, this is both the first meta‐analysis to determine CI and the first review of any type to address IR in paediatric HA‐RSV patients.

It is important to consider these results with care given potential factors affecting results. Efforts to minimise reviewer bias included the use of the Cochrane handbook, review by two independent authors with a third party acting to resolve disputes and direct contact with study authors for additional clarification as needed [17]. Despite these measures, additional potential sources of bias remain. The definition of nosocomial infection (Table 1) and institutional infection surveillance protocols varied widely between studies, which could introduce possible misclassification bias on the study level. There are also concerns for publication bias with Egger's regression test yielding a significant result (*t* = 4.3796, *p* = 0.0007). The relatively stable leave‐one‐out cross‐validation values (Figures S6 and S7) reduce concerns of meta‐analysis results being overly affected by any single study, enhancing the reliability of our results. Nevertheless, while it does support the consistency of the findings, it does not fully eliminate the possibility of systematic biases affecting the meta‐analysis, underscoring the need for cautious interpretation.

A key finding is the markedly higher MR of HA‐RSV compared to CA‐RSV. The MR of 11.34 deaths per 1000 person‐years found in our analysis is substantially higher than those in CA‐RSV meta‐analyses (6.21 deaths per 1000 children in Stein et al. [2017] and 5 in‐hospital deaths per 1000 children per year in Duan et a. [2023]) [18, 19]. Similarly, the CFR of 13.30% far exceeds the 0%–1.7% CA‐RSV CFR found in Bylsma et al. (2022) [20]. Additionally, while the CI of HA‐RSV among children with RSV was 15.57%, the RSV Gold registry—a global mortality registry of children under 5 years old who died with RSV infection—found that HA‐RSV–related deaths are composed 20% of the registry [10]. Thus, by all metrics, HA‐RSV seems to be disproportionately lethal compared to its CA counterpart.

The reason for this may be related to the host, the virus itself or both. The patient population affected by HA‐RSV is hospitalised patients, with many in the population having multiple comorbidities, such as respiratory failure, neuromuscular impairment and immunosuppression; in these patients, respiratory comorbidities are associated with increased risk of escalation (which could range from initiation of supplemental oxygen to increasing FiO_2_ during invasive ventilation) [21]. However, while this difference in comorbidities may be a confounding factor, helping to explain part of the differences in mortality, a study of matched HA‐RSV and CA‐RSV patients found children with HA‐RSV still had a substantially higher rate of respiratory escalation [22]. Thus, confounding by population differences is unlikely to fully explain this difference. A second potential reason lies with viral load. Several studies have demonstrated a link between higher viral loads and higher severity of RSV infection [23, 24]. Thus, as hospitalised RSV patients have higher viral loads than those in community environments [25], patients developing HA‐RSV were likely exposed to higher inocula than patients who developed CA‐RSV. This might contribute to a higher severity and mortality. Notably, the evidence of this theory is quite mixed. Some studies suggest lower viral loads may reflect sampling time, compartmentalisation to the lower respiratory tract or inadequate host response rather than lower inoculation [7]. Others demonstrated that higher viral loads early in infection could even protect against progression to hypoxic lower respiratory tract infection [26]. Thus, while higher exposure might contribute to the greater severity seen in HA‐RSV, further research is required to disentangle intrinsic viral pathogenicity from host vulnerability.

A major limitation in the meta‐analysis of this topic was the significant heterogeneity in the somewhat sparse literature on HA‐RSV. While the use of a random‐effects model rather than a fixed‐effects model appropriately mitigates statistical heterogeneity, the high degree of heterogeneity observed across nearly all outcome measures remains notable, reflecting differences in study populations, settings and methodologies. As outlined in Table 1, methodological inconsistencies in HA‐RSV definition between studies are a key source of study heterogeneity. Inconsistencies are twofold: the method to determine the start date of infection and the period in which an infection is considered hospital‐acquired. As seen in Table 1, some studies, such as Asner (2013), used the onset of signs and symptoms to determine the diagnosis date [27]; others, including Rodriguez‐Auad (2012), used laboratory confirmation of RSV infection to determine this [28]. Similarly, some studies like Asanathong (2017) considered any infection beginning at least 48 h post‐admission to be hospital‐acquired [29], whereas others like Fodha (2004) required at least 8 days post‐admission but also included infections beginning within 5 days of discharge to be hospital‐acquired [30]. Additionally, the method of RSV diagnosis varied, with techniques such as direct fluorescence antibody tests and rapid antigen tests being less sensitive than reverse transcription polymerase chain reaction; this can result in under‐ascertainment of true outcome estimates [31]. While this heterogeneity suggests the need to interpret pooled estimates cautiously, it also points to the need for standardisation of definitions and diagnostic protocols to improve comparability in future HA‐RSV research. To synthesise a sample definition, we calculated a pooled threshold for the period in which an infection would be considered hospital‐acquired using person‐time as weights to account for study size and exposure duration. Through this, HA‐RSV could be defined as RSV diagnosed at least 4 days following admission or within 1 day of discharge (exact values 97.35 [79.34–115.36] and 18.78 [1.30–36.27] h, respectively). Of note, the method of RSV diagnosis and the method to determine the start date cannot be standardised due to differences in study design and institution‐specific factors.

There is also a lack of literature reporting RSV transmission in the NICU and PICU, out with outbreak reports not included in the present analysis. Given that preterm infants, who comprise a disproportionately high proportion of the global RSV disease burdens, are often cared for in NICUs [32], the lack of NICU‐ and PICU‐specific data represents a critical gap. Likewise, few studies reported data on length of stay prior to HA‐RSV infection, an important metric for evaluating infection‐control measures in hospital settings. Due to the dearth of literature, data reporting RSV transmission by subgroup, such as age, or reporting on morbidity rates, such as ventilator usage, were likewise difficult in the current analysis. Limiting studies to patients only aged 3 years or less for IR analysis did demonstrate a higher rate of nosocomial infection (30.34 vs. 10.86 cases per 1000 person‐years) and was statistically significant. However, as this subgroup analysis was based only on three studies, it is difficult to attribute the difference solely to age with the current literature. Similarly, there were insufficient data to properly assess the impact of variable clinical phenotypes among variable patient groups, such as preterm infants, immunocompromised patients and those with underlying conditions. This could limit external validity outside of undifferentiated hospitalised patient populations. Such information in future studies could be of use to aid with further risk stratification of patients and to enable more targeted HA‐RSV prevention strategies.

While our review did not identify studies comparing rates of HA‐RSV before and after the SARS‐CoV‐2 pandemic, given global contact precautions and increased infection‐control protocols, such studies would be of interest. Previous articles have noted substantial decreases in RSV rates during the pandemic with resurgences following relaxation of rules surrounding non‐pharmacological interventions [33]. Likewise, recent innovations in RSV maternal vaccination and nirsevimab certainly have had impacts on RSV rates, and their impact on HA‐RSV rates is an area for future research [34].

Our meta‐analysis highlights the significant burden of HA‐RSV in hospitalised paediatric patients, underscoring the importance of recognising HA‐RSV as a serious and widespread issue in hospital settings, which accounts for a substantial portion of paediatric HAIs. While our analysis reveals notably higher mortality in HA‐RSV compared to CA‐RSV, further research is essential to fully understand the reason for this increased mortality. Despite limitations, our findings emphasise the urgent need for enhanced infection‐control measures, targeted prevention strategies and dedicated research to unravel the drivers of increased HA‐RSV mortality. Notably, infection‐control protocols for RSV such as hand washing and distance between patient beds have also been demonstrated to reduce HAIs secondary to other common viral causes of paediatric respiratory illness, including influenza and rhinovirus [35, 36, 37, 38]. This makes such measures all the more important. Further studies focusing on HA‐RSV outbreaks and the impact of surveillance and infection‐control techniques on prevention will likewise be impactful in advancing our understanding of HA‐RSV and improving outcomes for these children.

## Author Contributions

**Bhanu Wahi‐Singh:** investigation, methodology, software, formal analysis, data curation, validation, visualisation, writing – original draft, writing – review and editing. **Pia Wahi‐Singh:** investigation, methodology, data curation, validation, visualisation, writing – original draft, writing – review and editing. **Charlotte Lau:** conceptualisation, methodology, writing – review and editing, investigation. **Abigail Buckle:** conceptualisation, methodology, writing – review and editing, investigation. **Paolo Manzoni:** supervision, writing – original draft, writing – review and editing. **Harish Nair:** supervision, conceptualisation, investigation, methodology, project administration, validation, writing – original draft, writing – review and editing.

## Conflicts of Interest

Professor Paolo Manzoni reports speaker fees from Pfizer and AstraZeneca during the conduct of the study. Professor Harish Nair reports non‐financial support from Innovative Medicines Initiative, National Institute of Health and Care Research, AstraZeneca and MSD. The other authors declare no conflicts of interest.

## Peer Review

The peer review history for this article is available at https://www.webofscience.com/api/gateway/wos/peer‐review/10.1111/irv.70169.

## Supporting information


**Table S1:** EMBASE search strategy.
**Table S2:** CABI Global Health search strategy.
**Table S3:** MEDLINE Search Strategy.
**Table S4:** Cohort Study Joanna‐Briggs Institute Quality Assessment.
**Table S5:** Case–control study Joanna‐Briggs Institute quality assessment.
**Figure S1:** Forest plot of HA‐RSV IR for patients at ward‐level care.
**Figure S2:** Forest plot of HA‐RSV IR for patients in the NICU or PICU.
**Figure S3:** Forest plots of HA‐RSV CI for hospitalised patients.
**Figure S4:** Forest plots of HA‐RSV CI for patients with RSV.
**Figure S5:** Forest plots of HA‐RSV CI for hospitalised patients with an HAI.
**Figure S6:** Forest plot of HA‐RSV IR leave‐one‐out cross‐validation.
**Figure S7:** Forest plot of HA‐RSV MR leave‐one‐out cross‐validation.
**Figure S8:** Forest plot of HA‐RSV IR for high‐quality studies.
**Figure S9:** Forest plot of HA‐RSV MR for high‐quality studies

## Data Availability

The data that support the findings of this study are available from the corresponding author upon reasonable request.
