# Supplementary material for: The Epidemiology of Healthcare‐Acquired Respiratory Syncytial Virus Infection Among Hospitalised Paediatric Patients: a Systematic Review and Meta‐Analysis
Source: Influenza Other Respir Viruses. 2025 Oct 14;19(10):e70169. doi: 10.1111/irv.70169 (PMC12519416; doi:10.1111/irv.70169)
Supplement: Supplementary file 1 — Table S1: EMBASE search strategy. Table S2: CABI Global Health search strategy. Table S3: MEDLINE Search Strategy. Table S4: Cohort Study Joanna‐Briggs Institute Quality Assessment. Table S5: Case–control study Joanna‐Briggs Institute quality assessment. Figure S1: Forest plot of HA‐RSV IR for patients at ward‐level care. Figure S2: Forest plot of HA‐RSV IR for patients in the NICU or PICU. Figure S3: Forest plots of HA‐RSV CI for hospitalised patients. Figure S4: Forest plots of HA‐RSV CI for patients with RSV. Figure S5: Forest plots of HA‐RSV CI for hospitalised patients with an HAI. Figure S6: Forest plot of HA‐RSV IR leave‐one‐out cross‐validation. Figure S7: Forest plot of HA‐RSV MR leave‐one‐out cross‐validation. Figure S8: Forest plot of HA‐RSV IR for high‐quality studies. Figure S9: Forest plot of HA‐RSV MR for high‐quality studies [file IRV-19-e70169-s001.docx]

SUPPLEMENTARY MATERIALS

Search Strategies


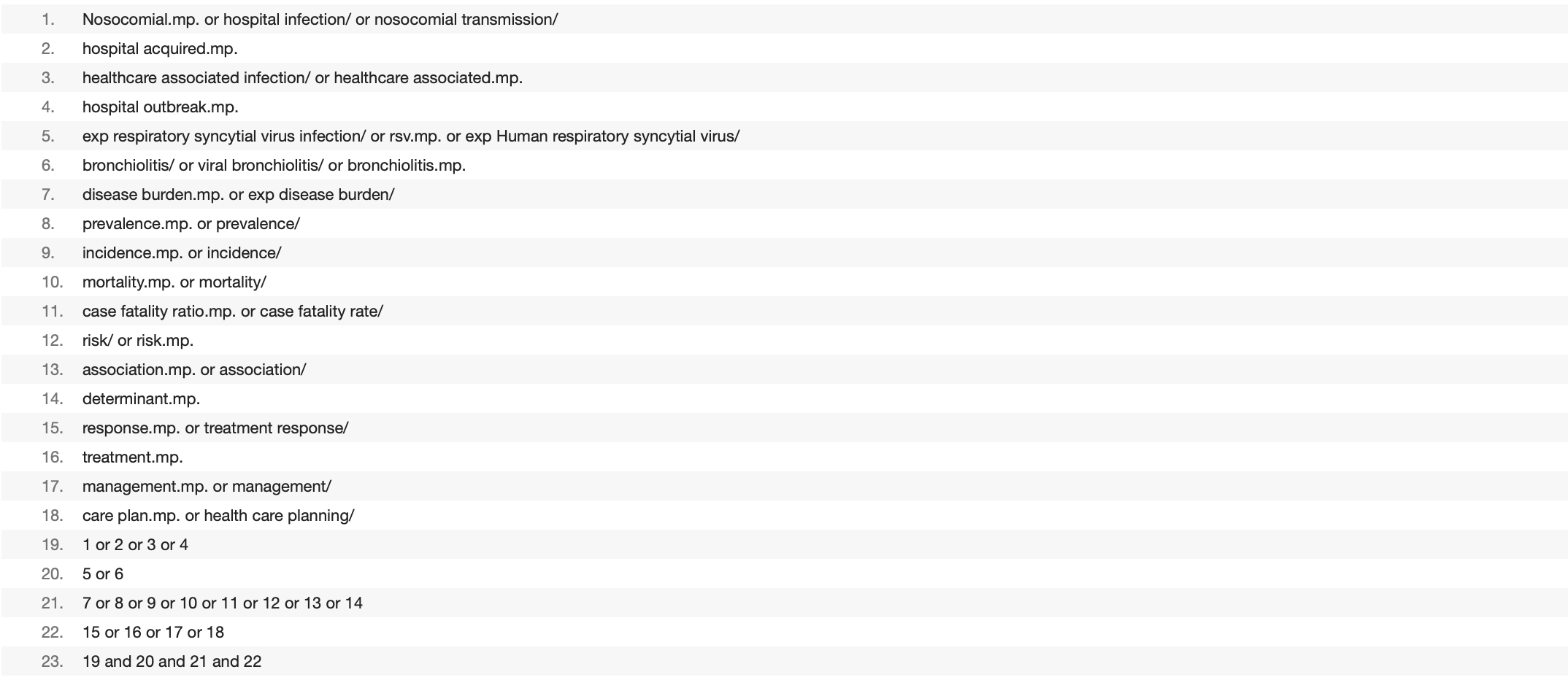


**Supplementary Table 1. Embase Search Strategy**

**
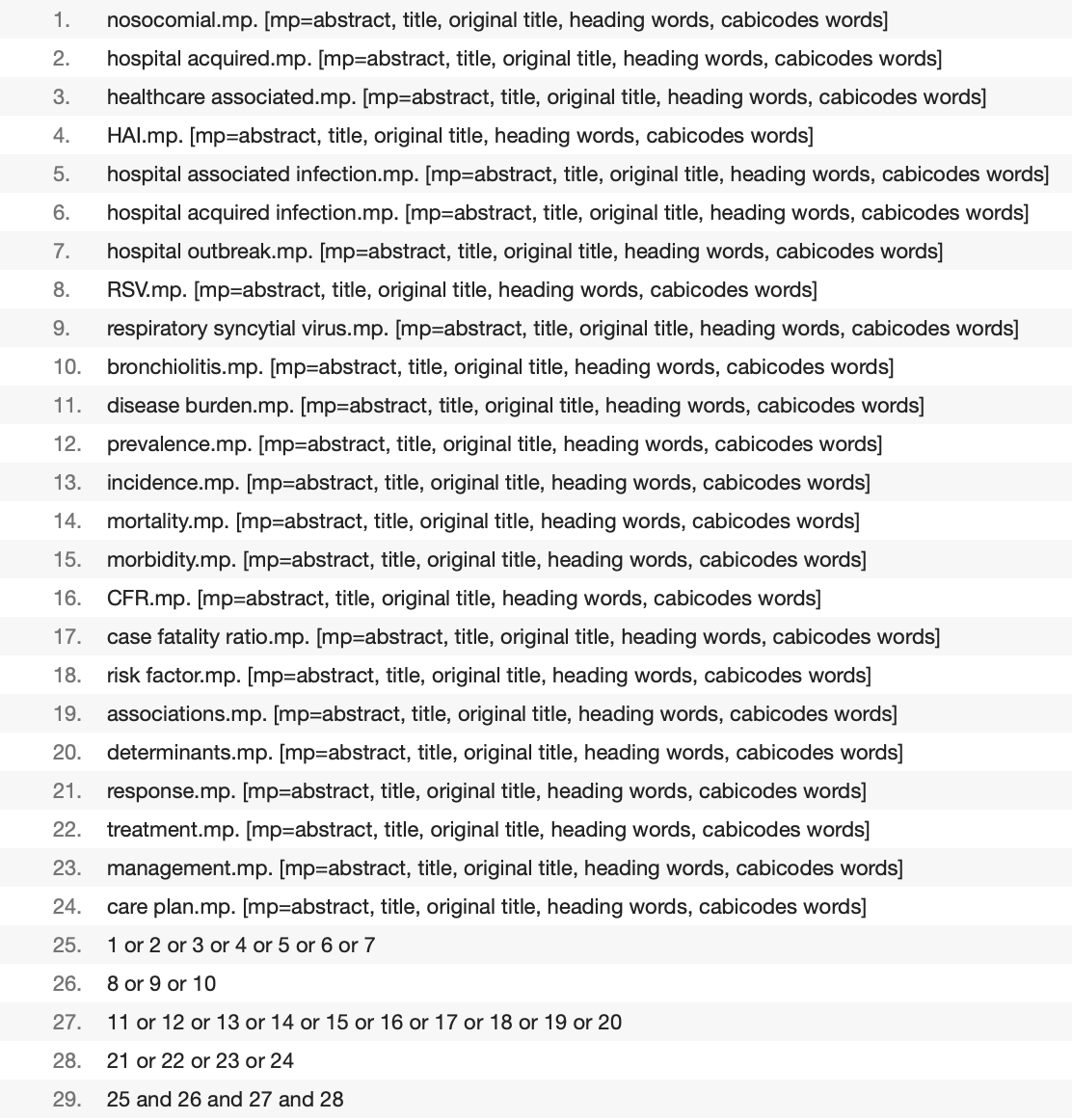
**

**Supplementary Table 2. CABI Global Health Search Strategy**

**
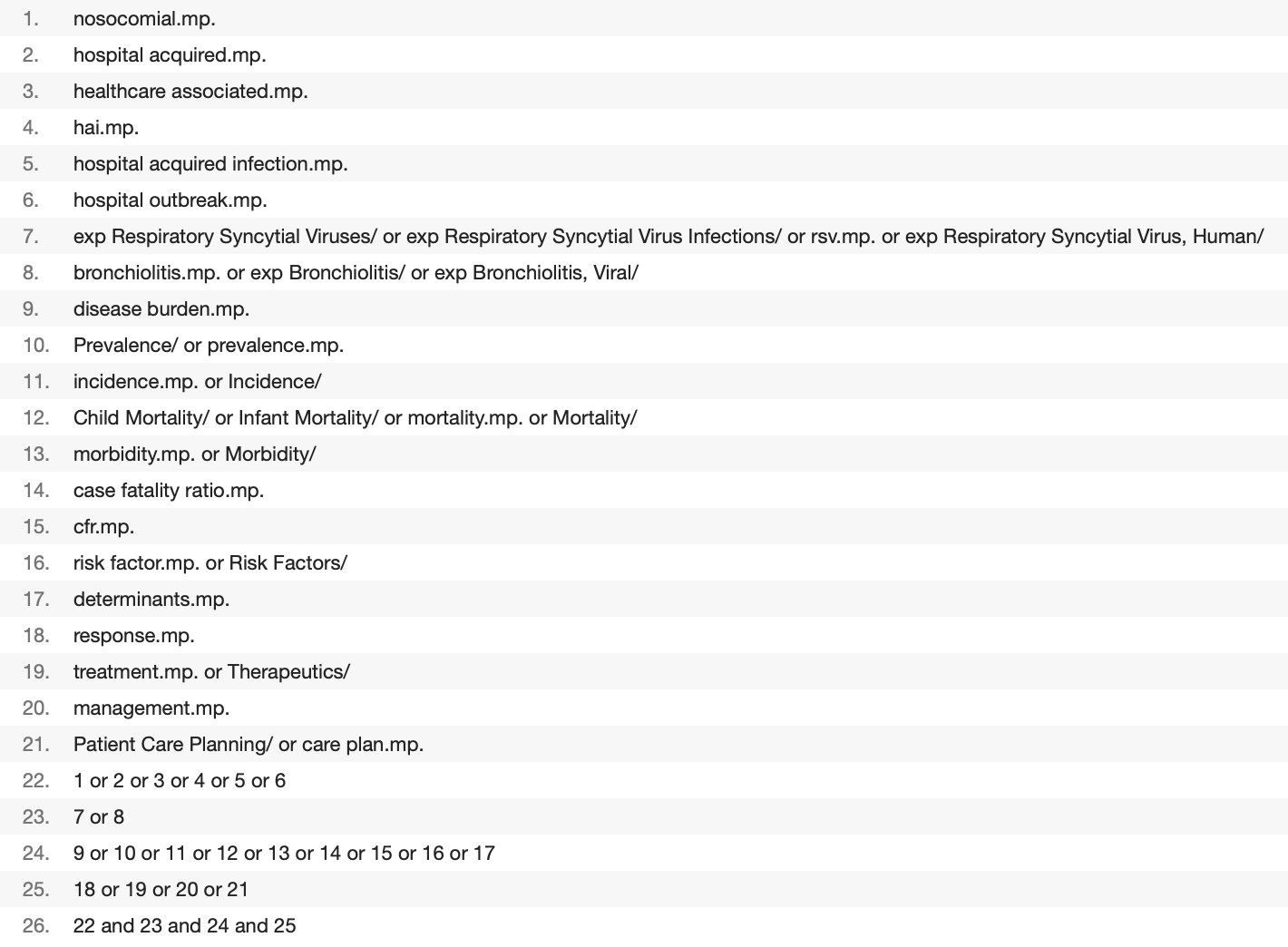
**

**Supplementary Table 3. MEDLINE Search Strategy**

Study Quality


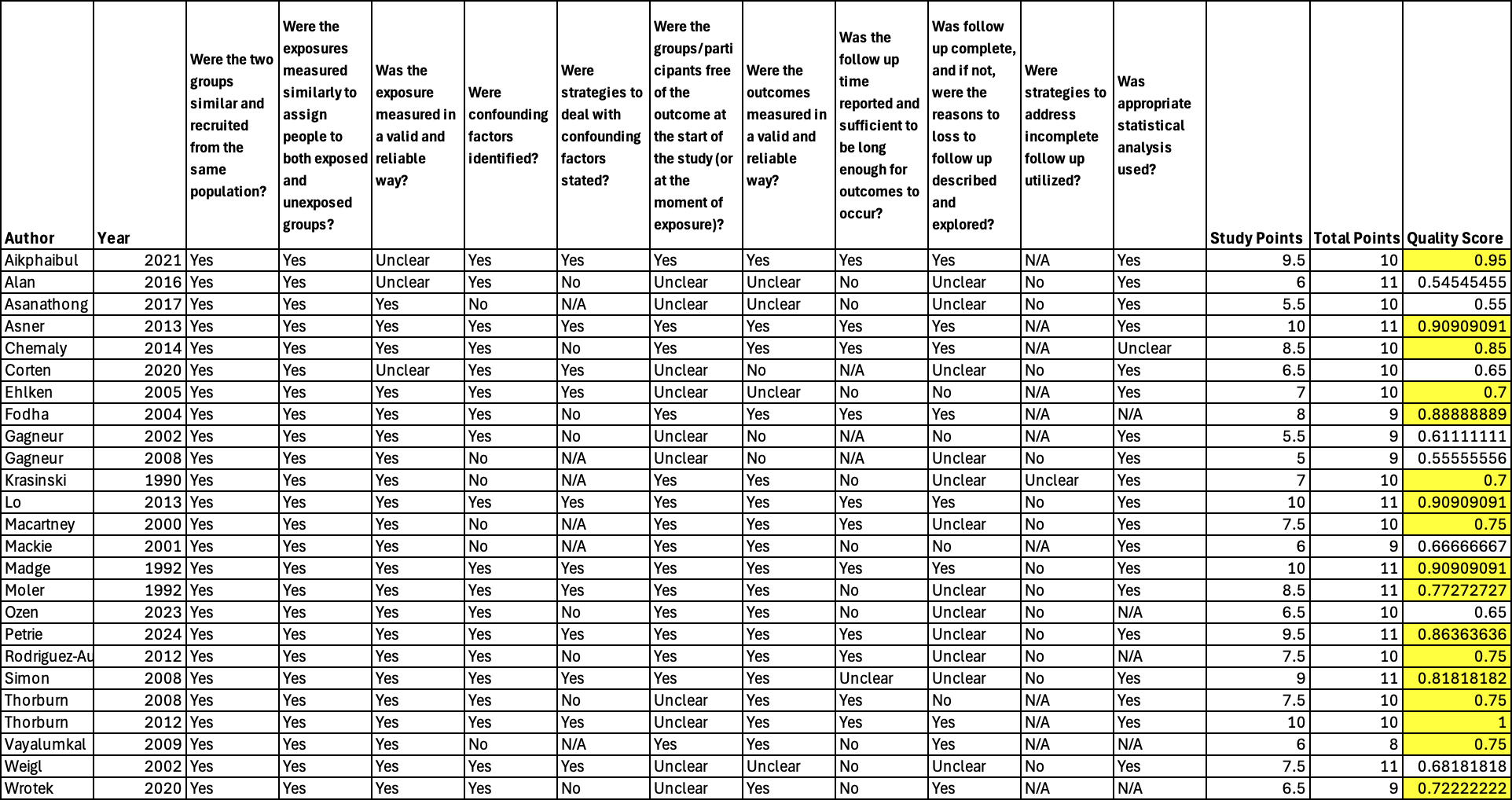


**Supplementary Table 4. Cohort Study Joanna-Briggs Institute Quality Assessment**


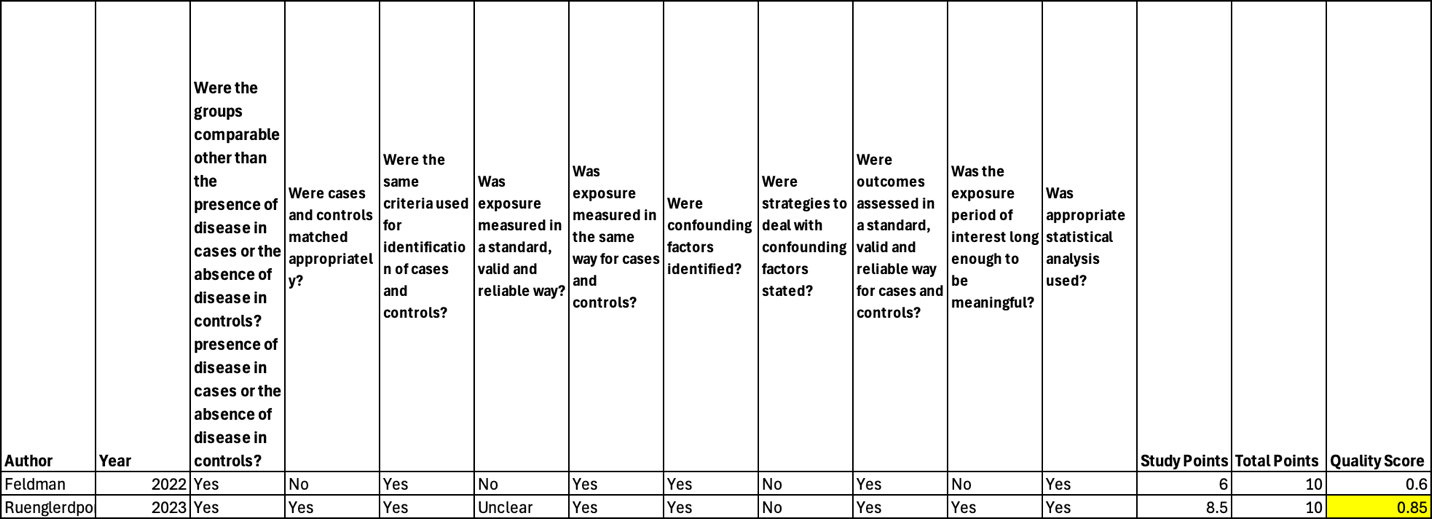


**Supplementary Table 5. Case-Control Study Joanna-Briggs Institute Quality Assessment**

Forest Plots for Incidence Rate by Study Setting


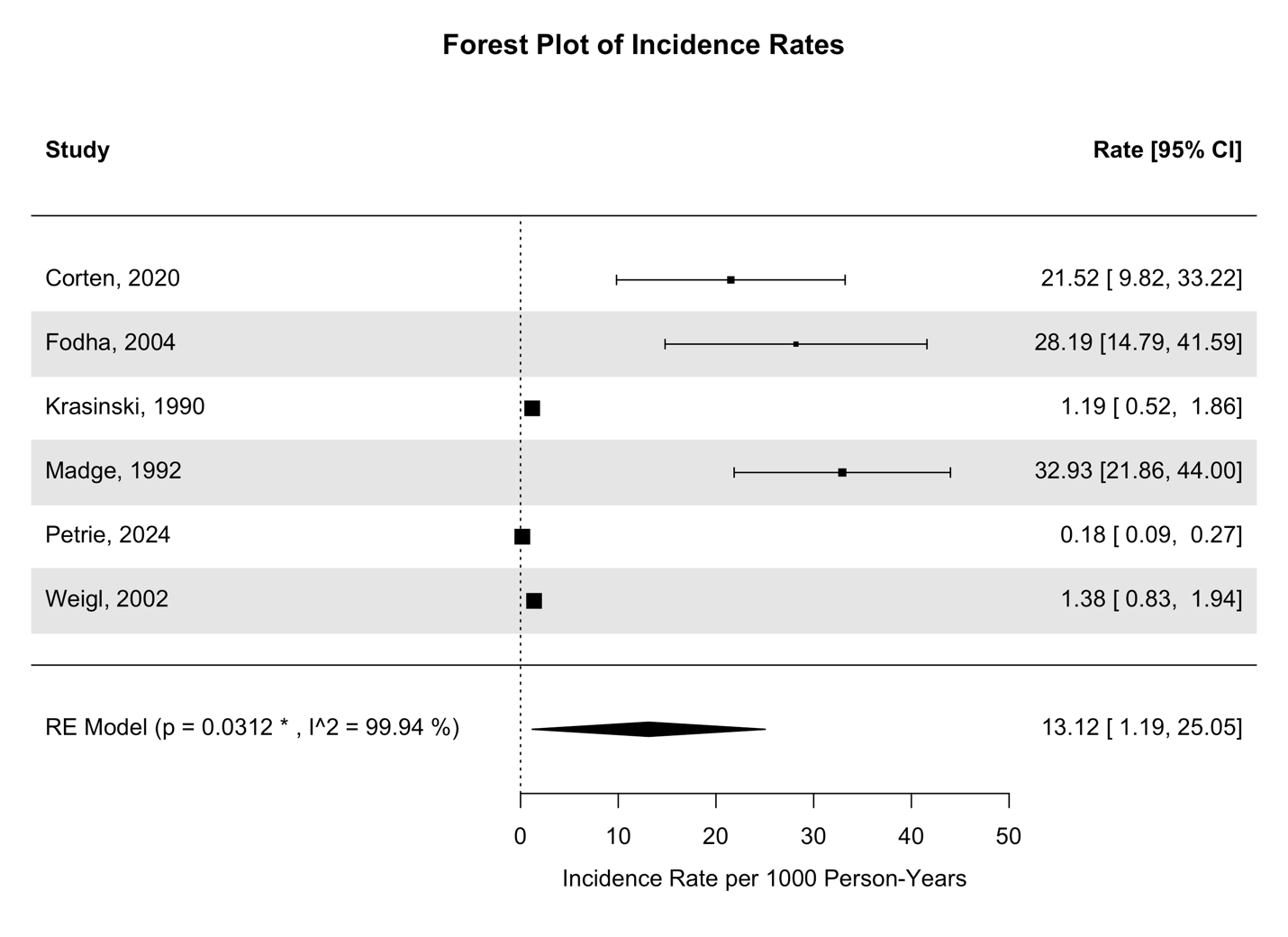


**Supplementary Figure 1. Forest Plot of HA-RSV IR for Patients at Ward-Level Care**


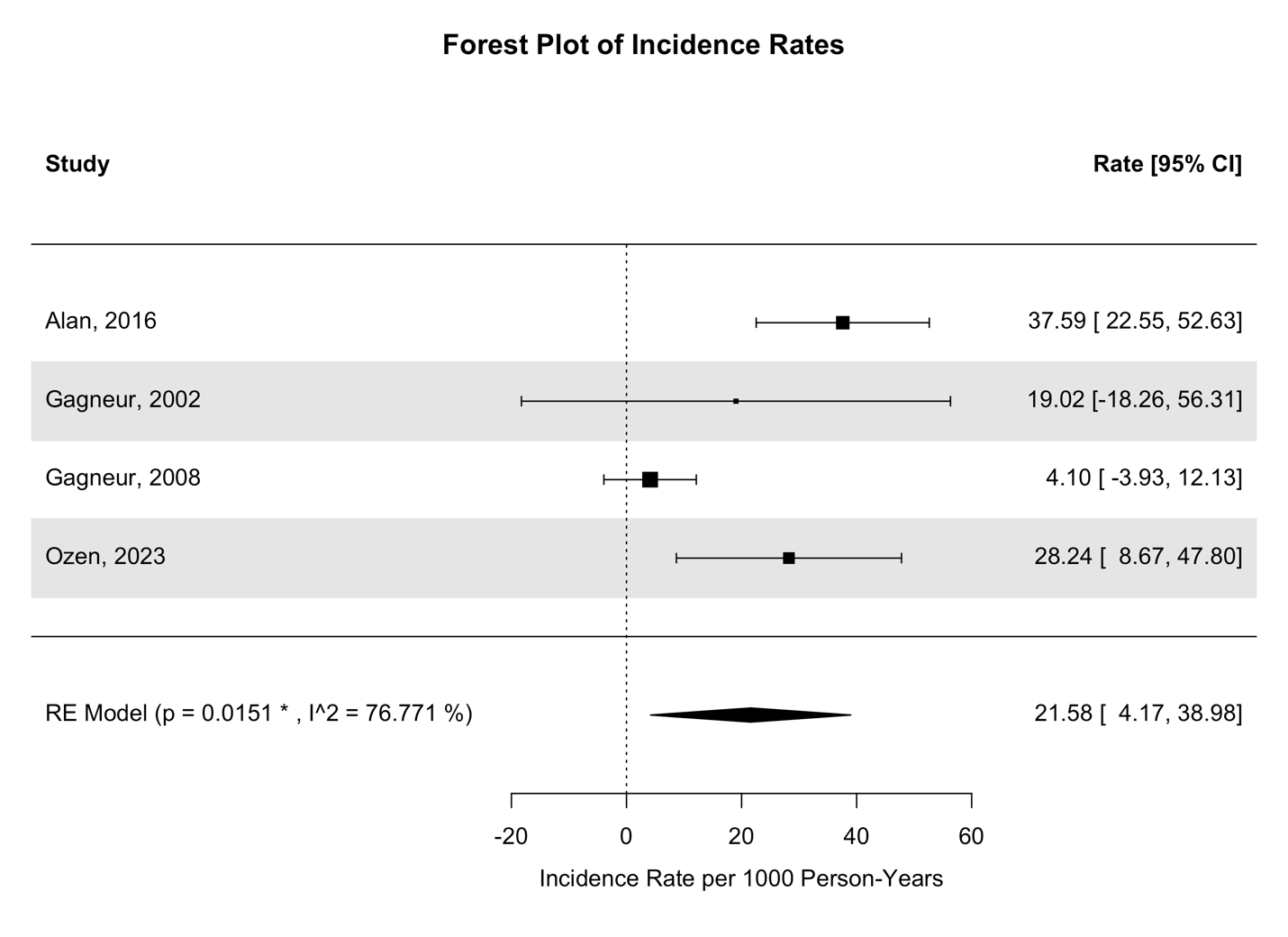


**Supplementary Figure 2. Forest Plot of HA-RSV IR for Patients in the NICU or PICU**

Forest Plots for Cumulative Incidence Analyses


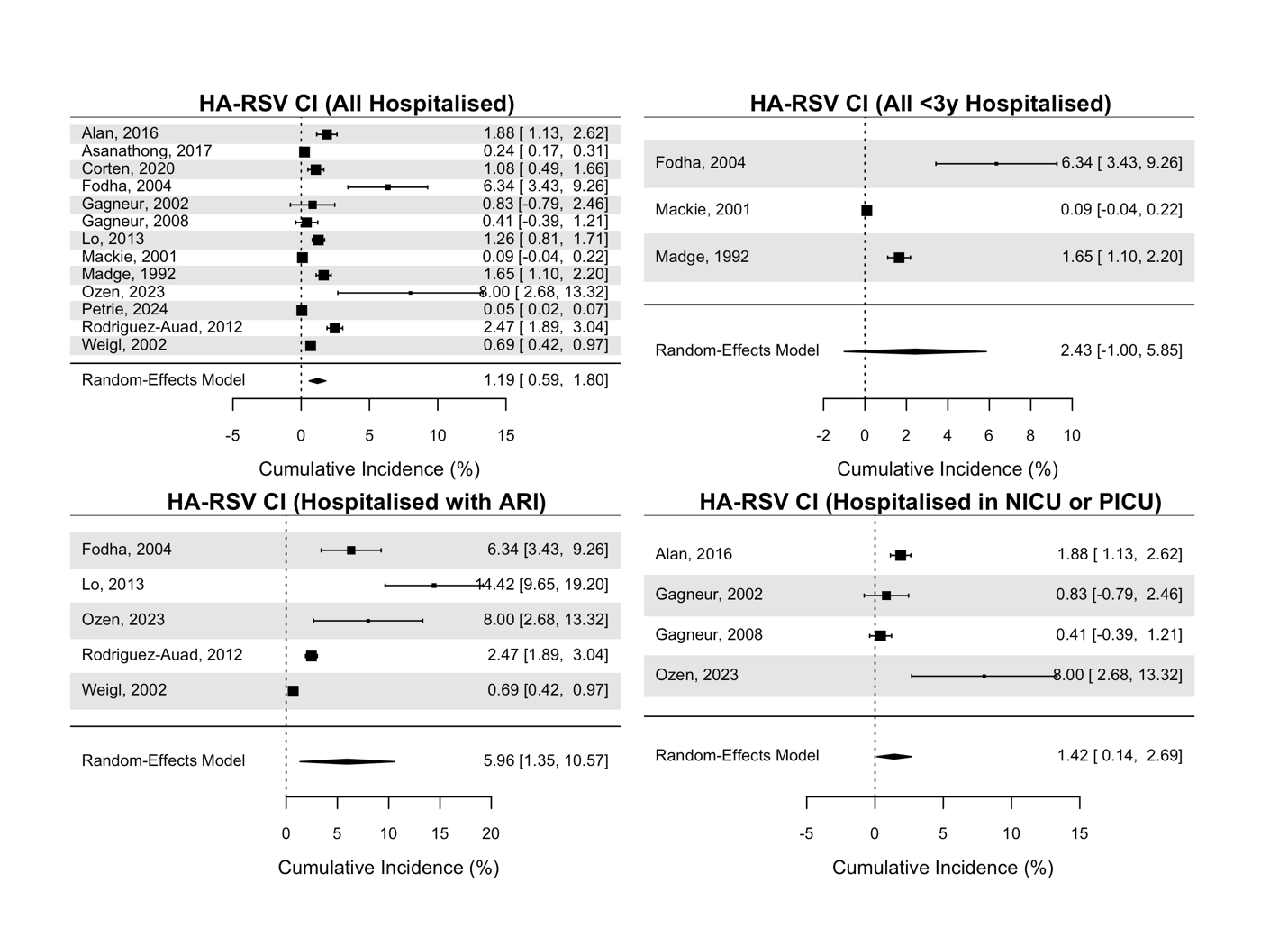


**Supplementary Figure 3. Forest Plots of HA-RSV CI for Hospitalised Patients**


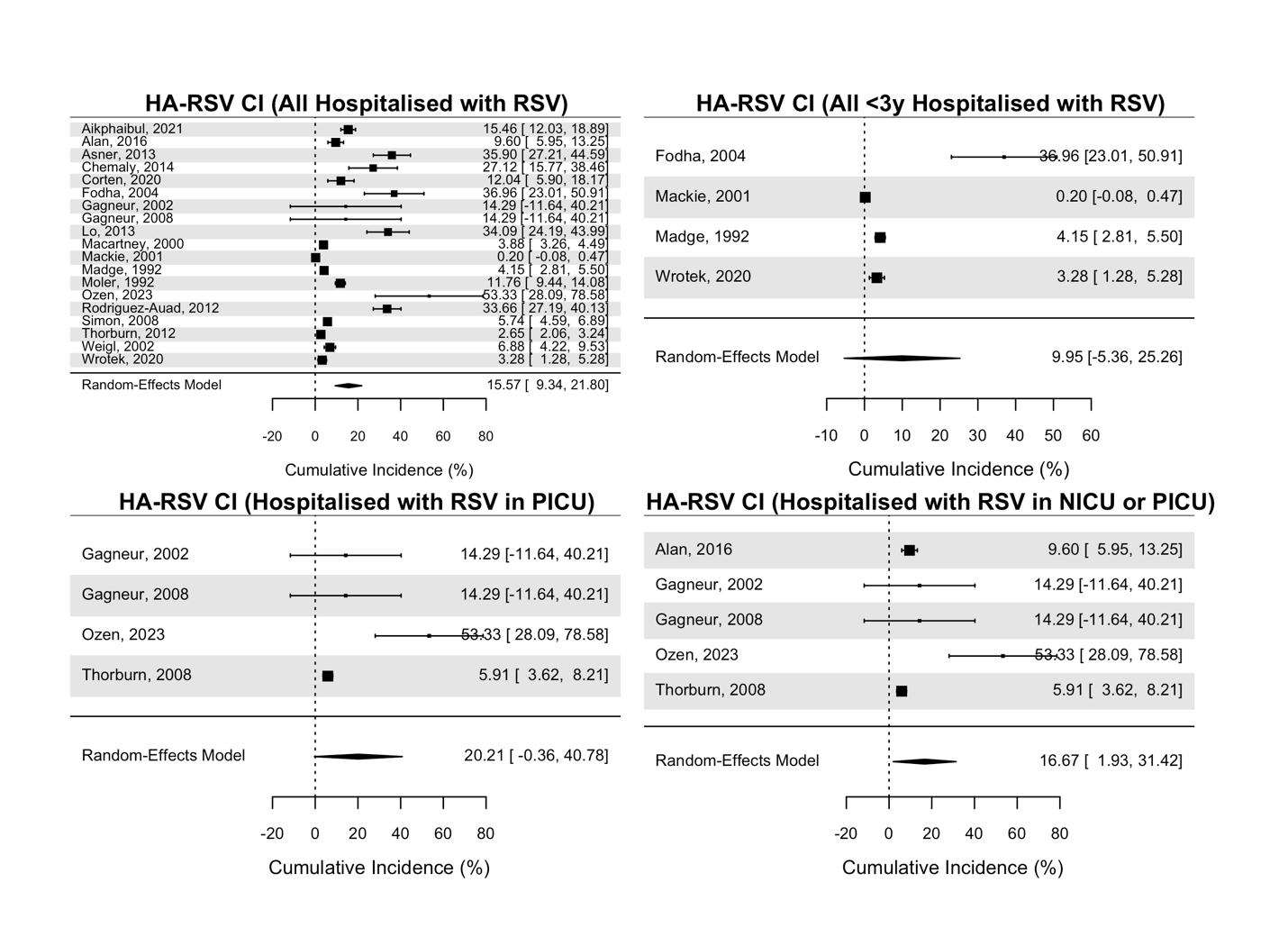


**Supplementary Figure 4. Forest Plots of HA-RSV CI for Patients with RSV**


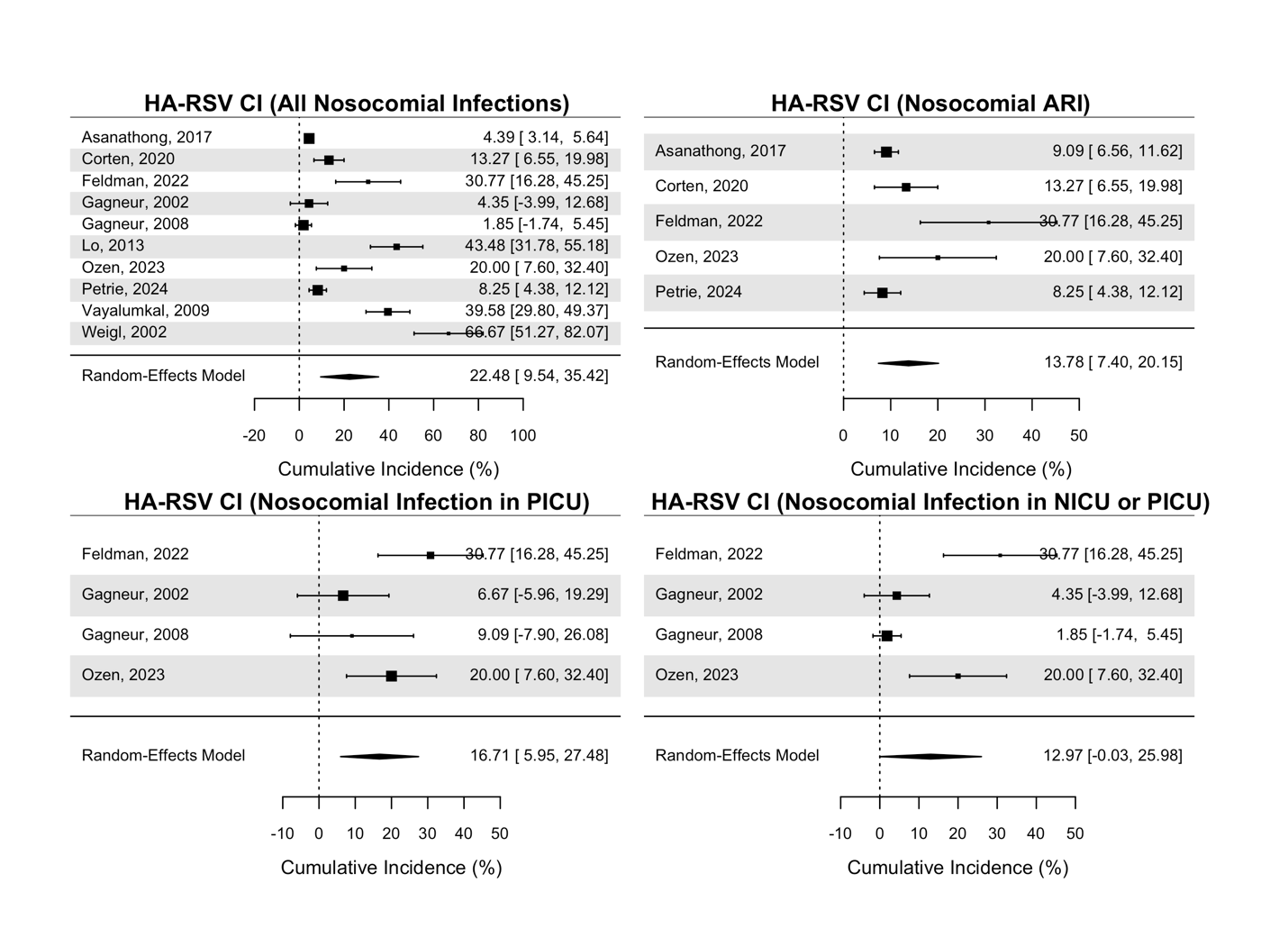
 **Supplementary Figure 5. Forest Plots of HA-RSV CI for Hospitalised Patients with an HAI**

Sensitivity Analyses


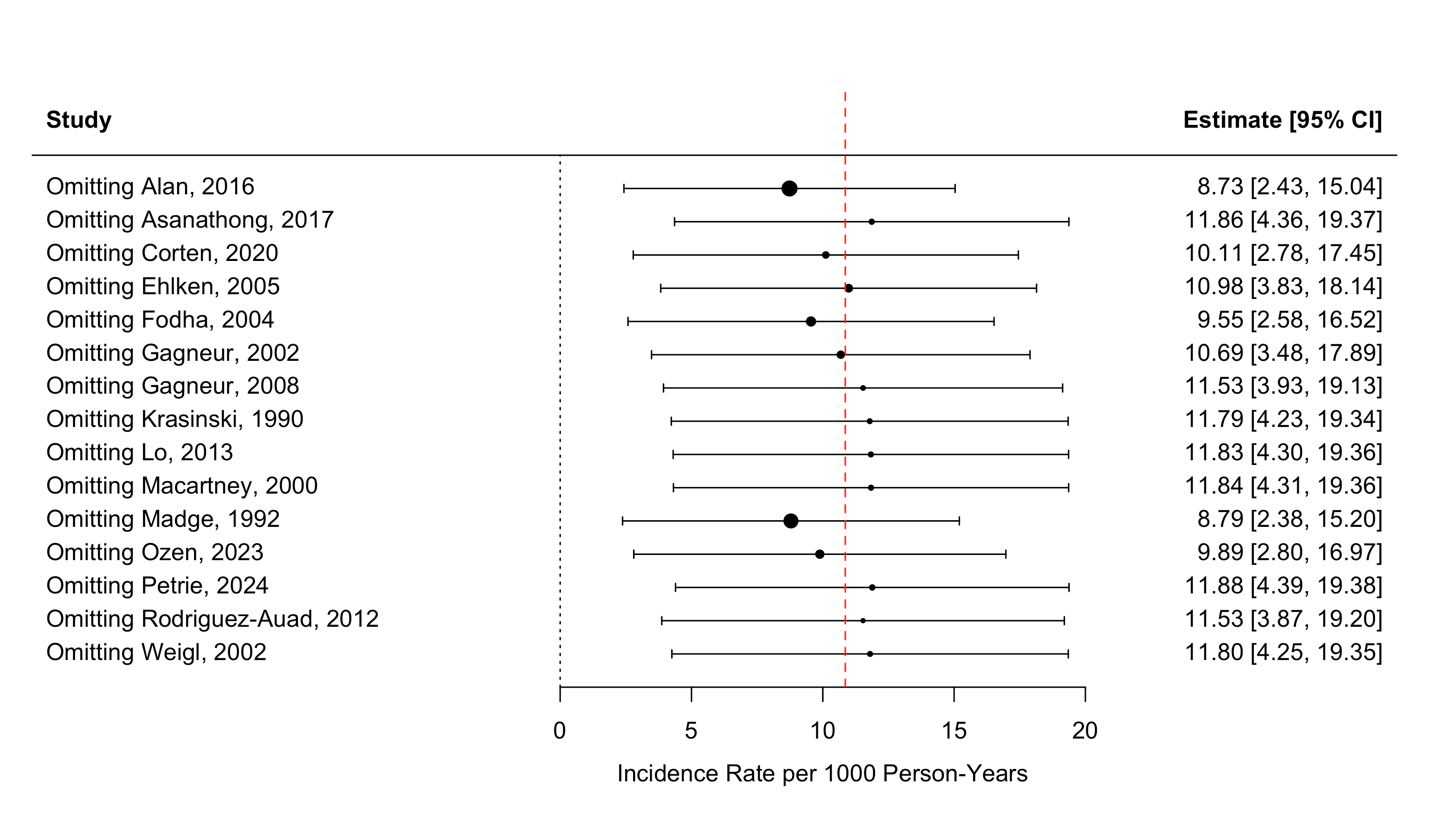
 **Supplementary Figure 6. Forest Plot of HA-RSV IR Leave-One-Out Cross-Validation**


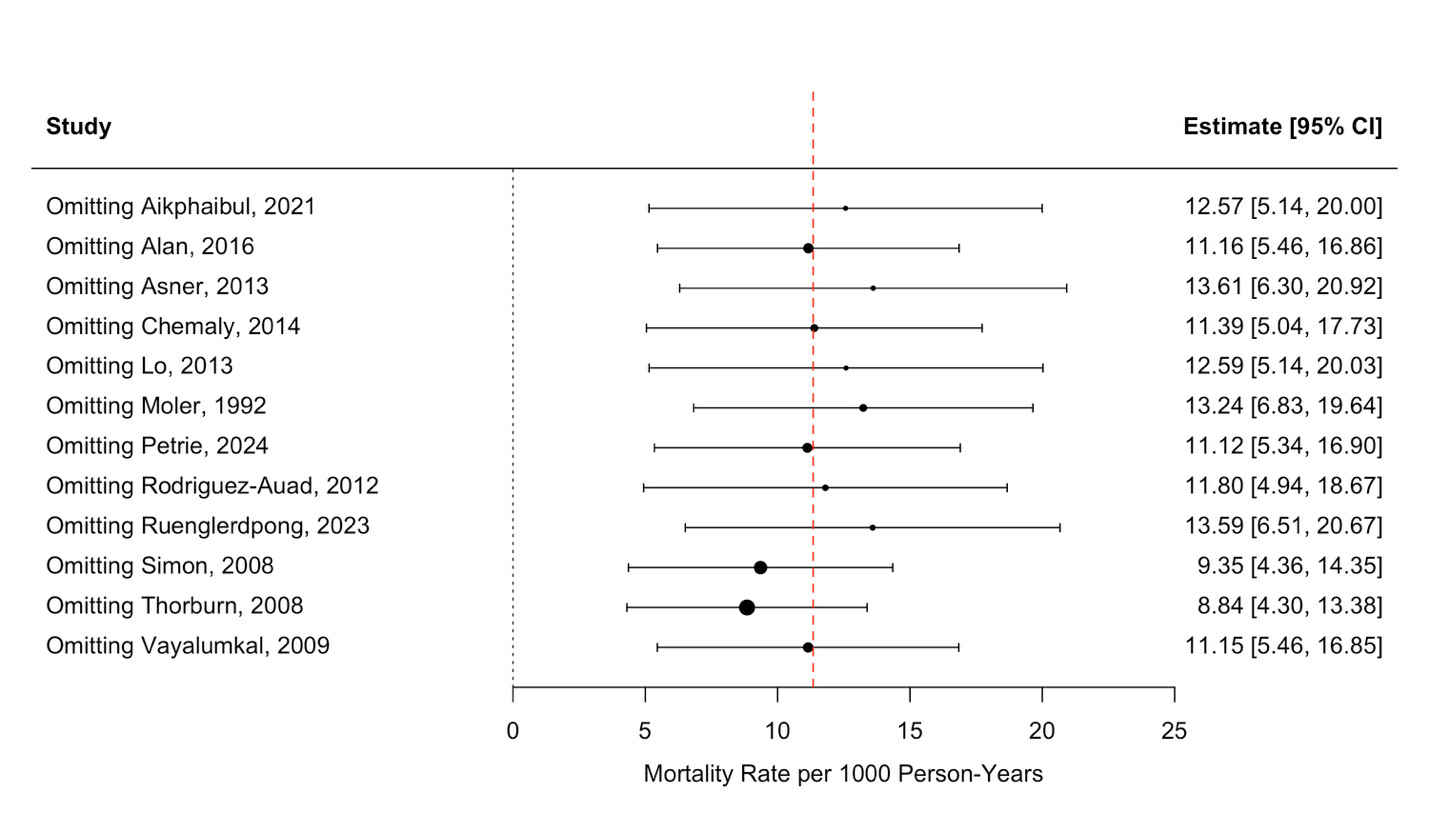


**Supplementary Figure 7. Forest Plot of HA-RSV MR Leave-One-Out Cross-Validation**

**
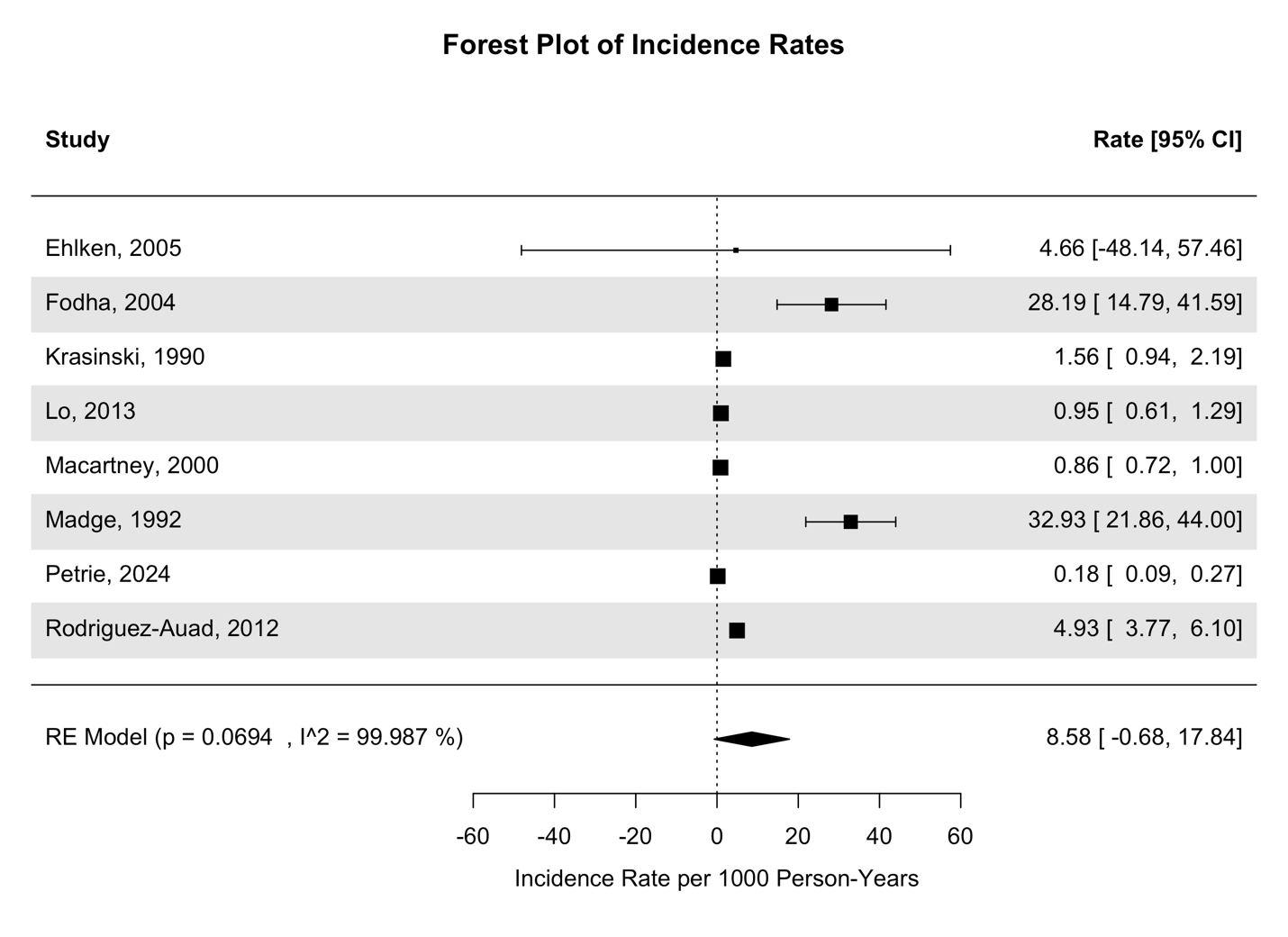
Supplementary Figure 8. Forest Plot of HA-RSV IR for High-Quality Studies
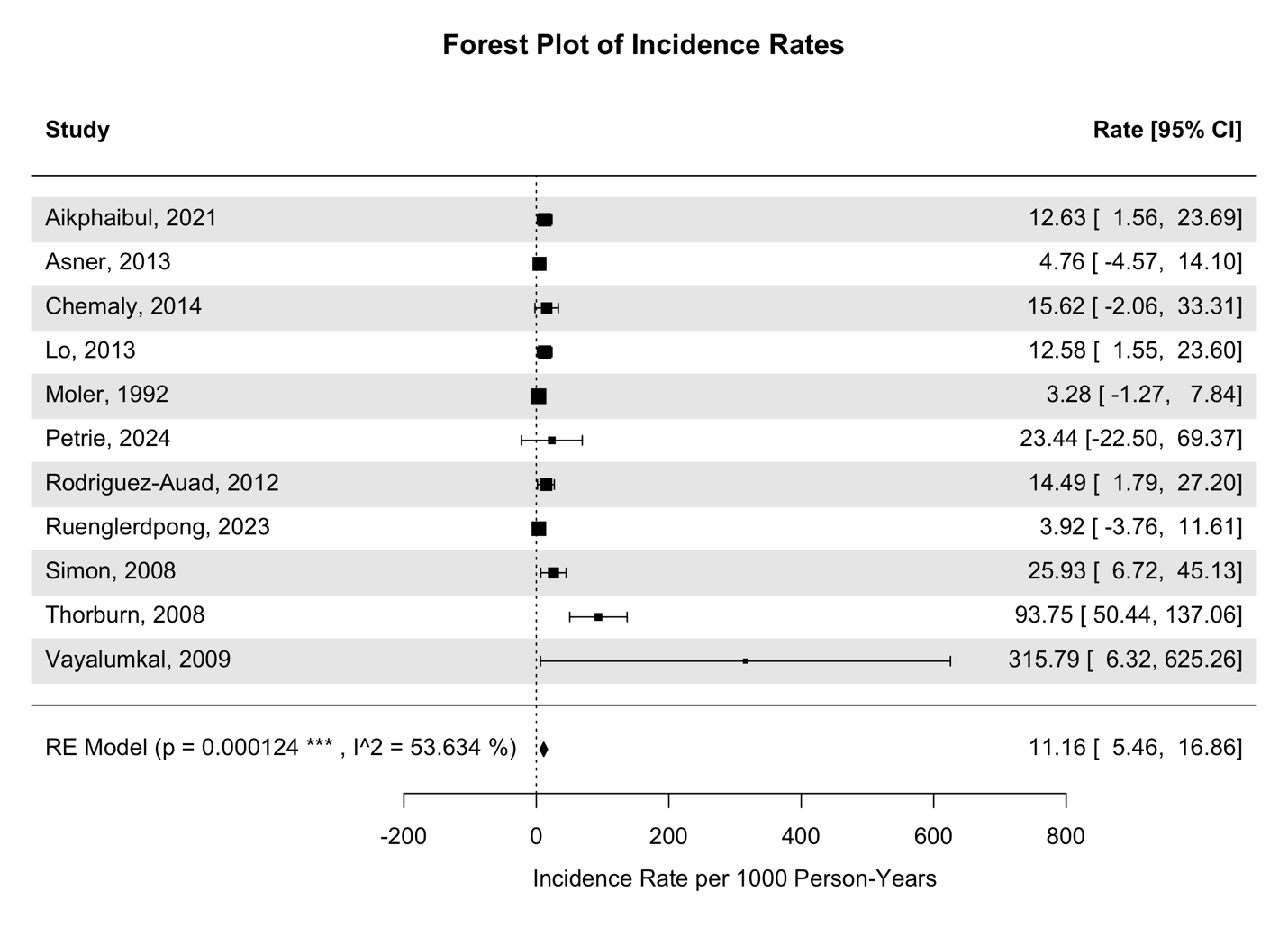
**

**Supplementary Figure 9. Forest Plot of HA-RSV MR for High-Quality Studies**
